# Antioxidant and immunomodulatory properties of polysaccharides from *Allanblackia floribunda* Oliv stem bark and *Chromolaena odorata* (L.) King and H.E. Robins leaves

**DOI:** 10.1186/s13104-015-1703-x

**Published:** 2015-12-09

**Authors:** Thaddée Boudjeko, Rosette Megnekou, Alice Louise Woguia, Francine Mediesse Kegne, Judith Emery Kanemoto Ngomoyogoli, Christiane Danielle Nounga Tchapoum, Olga Koum

**Affiliations:** Laboratory of Phytoprotection and Valorisation of Plants Resources, Biotechnology Centre-Nkolbisson, P.O. Box 3851, Messa, Yaounde, Cameroon; Department of Biochemistry, Faculty of Science, University of Yaounde I, P.O. Box 812, Yaounde, Cameroon; Department of Animal Biology and Physiology, Faculty of Science, University of Yaounde I, P.O. Box 812, Yaounde, Cameroon

**Keywords:** *Allanblackia floribunda*, *Chromolaena odorata*, Polysaccharides, Antioxidant activities, Immunomodulatory activities

## Abstract

**Background:**

Many plant polysaccharides have shown high antioxidant and immunostimulating properties and can be explored as novel molecules with biological properties that can potentially improve immune function. The objective of this work was to characterize soluble and cell wall polysaccharides isolated from the stem bark of *Allanblackia floribunda* and *Chromolaena odorata* leaves and to evaluate their antioxidant and immunomodulatory properties.

**Methods:**

Three polysaccharide fractions: soluble polysaccharides (PoS), pectins (Pec) and hemicelluloses (Hem) were extracted from *A. floribunda* stem bark and *C. odorata* leaves. These samples were analysed for their proteins, phenolic compounds and total sugar contents. The monosaccharide composition was determined by gas chromatography and arabinogalactan proteins content in PoS was evaluated by rocket electrophoresis. The in vitro antioxidant activities were evaluated by 1, 1-diphenyl-2-picryl hydrazyl (DPPH) and 2,2′-azino-bis-3-éthylbenzylthiazoline-6-sulphonic acid (ABTS) radical scavenging assays and ferrous ions chelating activity. Immunomodulatory activities were performed on the peripheral blood mononuclear cells (PBMCs) using proliferation and enzyme linked immunospot (ELISPOT) method to determine the production of an interferon-gamma.

**Results:**

The characterization of the various fractions showed varied metabolites in each plant. In PoS fractions, Ara and Gal were the major monosaccharides found, indicating that arabinogalactans are the primary macromolecules. Hem fractions contained predominantly Xyl and GalA for *A. floribunda* and Xyl (upto 80 %) for and *C. odorata*. *A. floribunda* Hem fraction and *C. odorata* PoS fraction showed significant DPPH and ABTS radical scavenging activities and immunostimulatory activity via stimulation of PBMC and production of IFN-γ in a dose-dependent manner.

**Conclusion:**

The results obtained from this study support the ethnomedicinal use of the stem bark of *A. floribunda* and leaves of *C. odorata.* Further research is necessary to have supporting evidence that the antioxidative and immunomodulative activities of these fractions are really connected to the polysaccharides and not polyphenols.

## Background

Polysaccharides are natural macromolecules consisting of multiple monosaccharides units. They represent a structurally diverse class of macromolecules that are widely distributed in nature and play an important role in controlling cell division, regulating cell growth and maintaining normal metabolism of living organisms. Polysaccharides of higher plants are a potential source of pharmacologically active compounds. Numerous studies have shown that polysaccharides isolated from medicinal plants could affect the immune responses both in vivo and in vitro and have the potential of being immunomodulators [1]. For example, polysaccharides such as lentinan, schizophyllan, and krestin have been used in clinical cancer therapies [2]. The biological activities of polysaccharides include antiviral, antitumor, immunostimulating, anti-inflammatory, anticomplementary, anticoagulant, hypoglycaemic, anti-mutagenic, anticancer, antioxidant and antitussive effects [2–4]. The discovery and evaluation of novel and safe polysaccharides from plants has become a popular research topic to detect functional foods or an alternative for the treatment of many diseases such as malaria, atherosclerosis and cancer inflammation, hypertension, wounds, scabies, rashes, abscesses, diseases associated with cellular degeneration and healing defects [4]. These various pathologies are generally evaluated in the cell through oxidative stress and inadequate immune response.

The phenomenon of oxidative stress is a state of imbalance between oxidized cellular components and antioxidants in favour of the oxidized components that can have serious consequences such as cancer and metabolic diseases. It has been shown that the parietal polysaccharides of some plants may have antioxidant activities; these include free radical scavengers [5], metal chelators [6], inhibitors of lipid peroxidation [7], reduction of DNA damage induced by H_2_O_2_/FeCl_2_ [8]. In addition, they exhibit hypoglycemic, hypolipidemic, immunomodulatory effects, stimulate macrophages and complement activities [9, 10] and increase the proliferation of lymphocytes [11].

*Allanblackia floribunda* Oliv (Clusiaceae) stem bark decoction is effective for the treatment of diseases such as hypertension, sexual weakness, and dysentery in the Centre and South Regions of Cameroon. Several studies on the aqueous extracts of the stem bark of *A. floribunda* showed that they have hypotensive activity in rats [12]. The oilseed cake improved the lipid profile on albino rats placed on a high fat diet [13]. Phytochemical data have reported the isolation of several molecules of the stem bark of *A. floribunda* (benzophenon, xanthons and biflavonoïds) [14]. These compounds have shown biological activities like cytotoxicity against cancerous cells, anti-inflammatory and antimicrobial effects [15].

*Chromolaena odorata* (L.) King and H.E. Robins. (Asteraceae) is a herbaceous plant with fast growth, native to South America. In folk medicine, the fresh leaves are used as cataplasms after wounds, burns and to stop bleeding. Its decoction is used to treat diarrhea, malaria and diabetes. Studies have shown that the leaves of *Chromolaenaodorata* are rich in sterols, polyterpenes, polyphenols, flavonoids, alkaloids, tannins and saponins [16]. The ethanolic extract of *Chromolaenaodorata* leaves has cytotoxic effect, anti-inflammatory and analgesic properties [16, 17]. Very little information is available on the antioxidant and immunomodulatory activities of the polysaccharides of *A. floribunda* and *C. odorata*.

Since decoction in water is the most common method of preparation and the most common routes of administration are oral and local application, we hypothesized that polysaccharides extracted from *A. floribunda* and *C. odorata* could have immunomodulatory properties and contribute to the therapeutic effects of extracts from these plants. This work is therefore to examine the total phenolic, protein, sugar and arabinogalactans proteins (AGPs) contents of soluble polysaccharides, pectic (Pec) and hemicellulosic (Hem) fractions of the stem bark of *A. floribunda* and leaves of *C. odorata* and to assess the antioxidant and immunomodulatory activities of these polysaccharides.

## Materials

### Plant material and preliminary treatment

*Allanblackia floribunda* stem bark (25633/SRF Cam) and *C*. *odorata* (n˚ 64190/SRF Cam) leaves were collected from the Centre region of Cameroon and identified at by the Cameroon National Herbarium. The plant material was chopped into small pieces and air-dried.

## Methods

### Extraction of soluble polysaccharides

Soluble polysaccharides (PoS) were extracted according to Schultz et al. [18]. 25 g of stem bark were ground into powder, put in 200 mL of distilled water and stirred overnight. The mixture was centrifuged at 4000*g* for 15 min. The supernatant was mixed with in four volumes of 95 % ethanol and kept at 4 °C. After 24 h, the precipitate was centrifuged at 4000*g* for 15 min. The residue collected was mixed with 5 mL of Tris–HCl buffer 50 mM, pH 8.0. The mixture was then dialyzed and freeze-dried.

### Extraction of cell wall polysaccharides

The wall was extracted according to the modified method of Ray et al. [19]. 5 g of plant powder were boiled in 100 mL of 85 % ethanol for 30 min. The mixture was centrifuged at 5000*g* for 15 min and the residue introduced into 100 mL of 90 % dimethyl sulfoxide and homogenized for 24 h at room temperature. After centrifugation under the same conditions, the residue was mixed with 100 mL of methanol and homogenized again for 24 h at room temperature. The mixture was centrifuged (5000*g* for 15 min), washed with acetone and the residue was dried at 45 °C.

To extract the pectic and hemicellulosic fractions, cell wall material (CWM) (1 g) was extracted twice with boiled ammonium oxalate at 0.5 % for 1 h, followed by incubation of the residue in 1 M KOH overnight at room temperature as described by Ray et al. [19]. All extracts were centrifuged and dialyzed against water. Alkaline extracts were acidified to pH 5 with acetic acid prior to dialysis. Each fraction extracted was gravimetrically analyzed.

### Quantification of proteins

Proteins were quantified using the Bradford [20] method. To each 1000 µL of polysaccharide extract was added 1 mL of Bradford reagent. The absorbance was measured at 595 nm using a UV-VIS 1605 Shimadzu spectrophotometer. BSA was used as the standard.

### Quantification of phenolic compounds

Phenolic compounds were estimated by using the Folin–Ciocalteu method [21]. 750 µL of extract solution (0.3 mg/mL) of soluble polysaccharides, pectin and hemicelluloses were added to 75 µL of Folin–Ciocalteu reagent. After 3 min, 750 µL of Na_2_CO_3_ (20 %) were added. The absorbance was measured at 760 nm using a UV-VIS 1605 Shimadzu spectrophotometer after 30 min in the dark. Phenolic compounds were calculated using ferulic acid as standard.

### Quantification of total sugars

The determination of the total amount of sugar was done according to the method of Dubois et al. [22] where the neutral monosaccharides were heated in acid medium and were transformed to dehydrated derivatives of furfural. Practically, 0.2 mL of sample was mixed with 0.2 mL of 5 % phenol. 1 mL of concentrated sulfuric acid was added quickly and stirred. The mixture was placed at 100 °C for 10 min during which it developed a yellow colour. The absorbance was read at 485 nm. The amount of sugar present and their levels were calculated using glucose as standard and expressed as µg equivalent of glucose per mg of dry polysaccharides.

### Analysis of monosaccharide composition

The monosaccharide composition of soluble polysaccharides, pectic and hemicellulosic fractions was determined as previously described by Ray et al. [19]. Neutral glycosyl composition was determined by gas chromatography of their alditol acetate and their trimethyl-silyl-glycosides derivatives respectively [23].

### Quantification of AGPs

AGPs quantification was done by glucosyl Yariv reagent binding in rocket gel electrophoresis. Briefly, electrophoresis was performed in 1 % agarose gel containing 15 µM Yariv reagent for AGP precipitation as described by Boudjeko et al. [24] in Tris–glycine buffer (25 mM Tris, 200 mM glycine, pH 8.4) for several hours, until the rockets were well developed. Known volume of PoS of *A. floribunda*, PoS of *C. odorata* and 7 μL of standard solution (AGP of gum arabic) (1 mg/mL) were deposited in each well. The concentration of AGP in the samples was estimated in relation to the peak area of gum arabic.

### 1,1-diphenyl-2-picryl hydrazyl (DPPH) free-radical-scavenging assay

The free radical scavenging activity of the polysaccharide fractions was measured in terms of their hydrogen donating or radical scavenging ability using the DPPH radical [8]. For the assay, 4000 µL of the fraction at different concentrations [50–600 µg/mL] were introduce into test tubes and 1000 µL of the freshly prepared solution of 400 µM DPPH in methanol were added. The mixture was stirred and left in the dark at 37 °C for 30 min. The absorbance was measured at 517 nm using a UV-VIS 1605 Shimadzu spectrophotometer. Ascorbic acid (Asc) and Catechin (Cat) were used as the positive control.

### Acide 2,2′-azino-bis-3-éthylbenzylthiazoline-6-sulphonique (ABTS) free-radical-scavenging assay

The method described by Re et al. [25] was adopted. To each 20 µL of different extracts (0.5, 1, 2, 3 and 4 mg/mL) concentration was added 1 mL of ABTS reagent. The mixture was agitated and kept in the dark for 30 min. The absorbance was measured at 734 nm using UV-VIS 1605 Shimadzu spectrophotometer. Cat was used as the positive control.

### Chelating capability for ferrous ions

The method of Dinis et al. [26] was used to determine the ferrous ion chelating ability. 1000 µL of different extracts [100–500 µg/mL] were mixed with 100 µL of FeCl_2_ (2 mM) and left in the dark for 1 min. The mixture was added to 200 µL of ferrozine (5 mM). After 10 min at room temperature, the absorbance of the mixture was read at 562 nm against a blank (distilled water) using a UV-VIS 1605 Shimadzu spectrophotometer. EDTA was used as the positive control.

### Immunomodulatory activity of polysaccharide fractions

#### Preparation of human peripheral blood mononuclear cells (PBMCs)

Authorization for the use of human blood in this study was obtained from the Institutional Review Board of the Biotechnology Centre (University of Yaounde 1, Cameroon). Signed informed written consent was obtained from each enrolled person. The blood was collected from men and women between 23 and 35 years and diluted with 10 mL of incomplete Roswell Park Memorial Institute (RPMI-1640) medium. 15 mL of this mixture was added to Ficollin 2/3–1/3 (v/v) and centrifuged at 10,000*g* for 25 min. PBMCs suspended in 4 mL of incomplete RPMI 1640 medium was washed and centrifuged at 8000*g*. After separation, PBMCs was finally diluted in complete RPMI 1640 medium containing type AB human serum, Hepes, 200 mM l-glutamine and 50 mg/mL Gentamicin [27] and counted with lazarrus.

### Cell proliferation measurement and INF-γ production by enzyme linked immunospot technique (ELISPOT)

The assays were carried out according to the procedures in the ELISPOT kit manual. The INF-γ produced by PBMCs sensitizes on coat plate to form an immunocomplex. This immunocomplex was revealed by an insoluble chromogene subtrate, in which the precipitation localized generate ruddy stain [28]. The human PBMCs culture was incubated for 16 h at 37 °C with 5 % CO_2_ at 90 % RH with different polysaccharide-enriched fractions at 100 µg/mL and 200 µg/mL and subjected to test the production of INF-γ cytokine.

### Statistical analysis

Results were expressed as mean ± SD of three parallel measurements; ANOVA followed by the Dunett test was used to test the significant difference using GraphPad Instat 3.05 software. The level of significance was set at *p* < 0.05 and the graphical representations were designed using Microsoft Excel 2007.

## Results

### Yield of polysaccharide fractions, total protein and phenolic compound levels

**Fig. 1:**
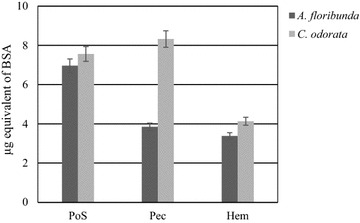
Total protein content of polysaccharide isolate from *Allanblackia floribunda* stem bark and *Chromolaena odorata* leaves by the Bradford method

**Fig. 2:**
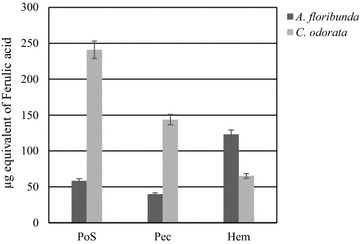
Total phenolic content of polysaccharide isolate from *Allanblackia floribunda* stem bark and *Chromolaena odorata* leaves by the Folin–Ciocalteu method

Gravimetric analysis of lyophilized fractions after sequential fractionation (Table 1) yielded significant differences between *A. floribunda* stem bark and *C. odorata* leaves in water-soluble fractions, ammonium oxalate fractions and 1 M KOH fractions. Water extracted more material (water-soluble-polysaccharides fractions) from *C. odorata* (2.95 % Dry weight (Dw)) than *A. floribunda* (1.9 % Dw) sample. On the other hand, ammonium oxalate extracted more material in *A. floribunda* (5.5 % of CWM) than in *C. odorata* (2.36 % of CWM) (Table 1). The partial characterization of total protein and phenolic contents showed that all fractions were mostly a complex of proteins (Fig. 1) and phenolic compounds (Fig. 2). Quantification of proteins content in polysaccharide fractions showed that PoS of *C. odorata* and Pec of *A. floribunda* present the higher content with 7.56 ± 0.03 and 8.32 ± 0.14 µg BSA equivalent respectively (Fig. 1). For the phenolic compounds, their content were higher in the Hem fraction (151.95 ± 14.42 µg equivalent of ferulic acid/mg Dw) of *A. floribunda* and in the PoS fraction (241.04 ± 20.78 µg equivalent of ferulic acid/mg Dw) of *C. odorata* (Fig. 2).

**Table 1:** Yield of lyophilized polysaccharide fractions isolated from *A. floribunda* and *C. odorata*

Fractions	*A. floribunda*	*C. odorata*
Water-soluble fractions	1.9 ± 0.04	2.95 ± 0.07
Cell wall material	40.65 ± 3.2	34.00 ± 2.8
Ammonium oxalate fraction	5.5 ± 0.8	2.36 ± 0.6
KOH 1 M fraction	2.5 ± 0.2	7.55 ± 0.5

### Total sugar content, AGPs content and monosaccharide composition

**Fig. 3:**
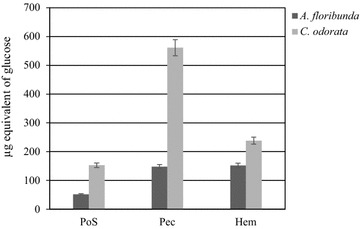
Total sugar content of polysaccharide isolate from *Allanblackia floribunda* stem bark and *Chromolaena odorata* leaves by the phenol–sulfuric acid method

**Fig. 4:**
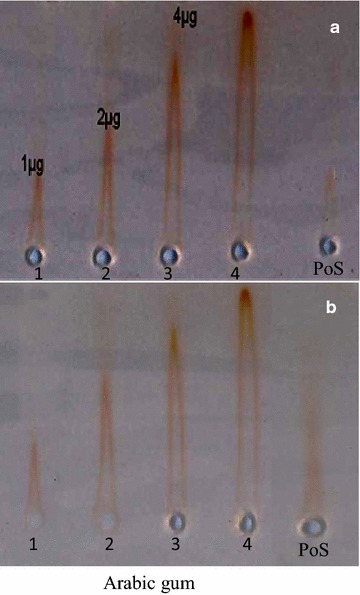
Quantification of total arabinogalactan–proteins in soluble polysaccharide fractions of *Allanblackia floribunda* stem bark (**a**) and *Chromolaena odorata* leaves (**b**) by rocket electrophoresis. Arabic gum AGP (1 mg/mL) was used as standard

In the water-soluble fractions, the sugar content estimated by the phenol–sulfuric acid method [23], was much lower in *A. floribunda* than in *C. odorata* (Fig. 3). This low levels of total sugar content in water-soluble fraction suggested that this fraction could be richer in water-soluble proteins or in glycoproteins such as AGPs. The stem bark of *A. floribunda* had 147.54 ± 4.38 and 151.95 ± 1.97 µg/mg of dry polysaccharides for Pec and Hem respectively, while Pec and Hem of *C. odorata* leaves contained 561.17 ± 23.15 and 238.12 ± 20.5 µg/mg of dry polysaccharides respectively (Fig. 3). The total AGPs estimated by rocket electrophoresis on agarose gel showed that PoS of *A. floribunda* stem bark contained approximately 200.7 µg/g of Dw. While, PoS of *C. odorata* contained approximately 25.3 µg/g of Dw (Fig. 4).

**Fig. 5:**
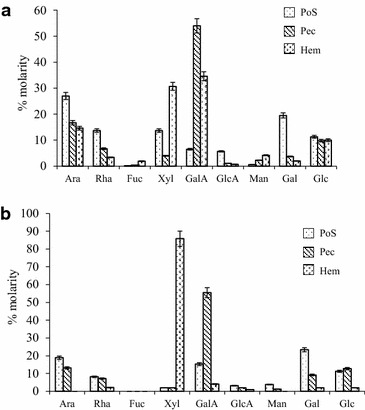
Monosaccharide molar composition of polysaccharide isolate from *Allanblackia floribunda *stem bark (**a**) and *Chromolaena odorata* leaves (**b**)

The monosaccharide composition is shown on Fig. 5. In *A. floribunda* (Fig. 5a), the PoS is mainly composed of Ara (27 %) and Gal (19.3 %). The high level of Xyl (30.7 %) and Ara (14.6 %), in the Hem of *A. floribunda*, suggests the presence of arabinoxylan type hemicellulose. Pec contained more than 50 % of GalA (Fig. 5a). In *C. odorata* leaves, PoS was mainly composed of Gal (23.4 %), Ara (18.9 %) and GalA (15.3 %); Pec was rich in GalA (55.5 %), Ara (13.2 %) and Glu (12.8 %) and Hem mainly contained Xyl (85.8 %) (Fig. 5b).

### DPPH and ABTS radical scavenging activities

**Fig. 6:**
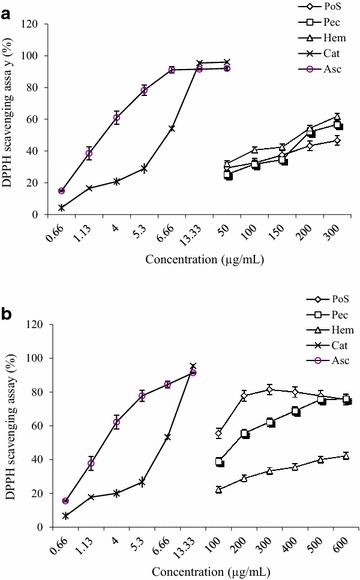
DPPH-free radical scavenging activity (%) of polysaccharide isolate from *Allanblackia floribunda* stem bark (**a**) and *Chromolaena odorata* leaves (**b**). Asc: Ascorbic acid; Cat: Catechin

**Fig. 7:**
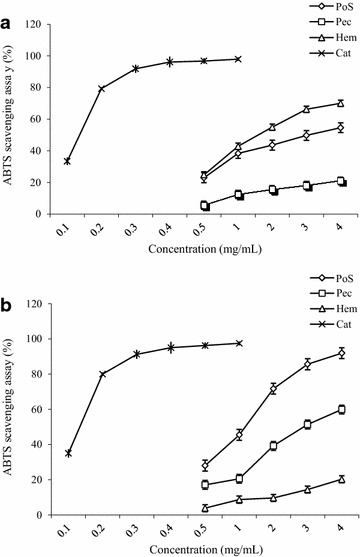
ABTS scavenging activity (%) of polysaccharide isolate from *Allanblackia floribunda* stem bark (**a**) and *Chromolaena odorata* leaves (**b**). Cat: Catechin

The DPPH free radical, a stable radical with purple color with a maximum absorption at 517 nm, has been widely used as a tool to evaluate the free radical scavenging activities of antioxidants [8, 13]. When DPPH encounters antioxidant scavengers, its purple colour rapidly fades because it is changed to the non-radical form DPPH-H. On the basis of this principle, the scavenging effects of the polysaccharide fractions on the DPPH radical were measured and the results are shown in Fig. 6. The scavenging activities of polysaccharides fractions increased significantly with the increase in sample concentration ranging from 50 to 300 µg/mL for *A. floribunda* and 100 to 600 µg/mL for *C. odorata*. In relation to the DPPH inhibition, Hem of *A. floribunda* presented the best activity with 61.84 ± 1.3 % at 300 µg/mL (Fig. 6a). For *C. odorata* polysaccharide fractions, at the concentration of 300 µg/mL, the scavenging activities were 27.70, 59.91 and 23.20 % for PoS, Pec and Hem respectively (Fig. 6b). The same observations were made with ABTS radical scavenging activity, where Hem *A. floribunda* presented the best activity with 70.01 ± 0.16 % at 4 mg/mL (Fig. 7a). However, for *C. odorata*, the best activity was obtained with PoS (91.91 ± 0.9 % at 4 mg/mL) (Fig. 7b).

### Ferrous ion chelating ability

**Fig. 8:**
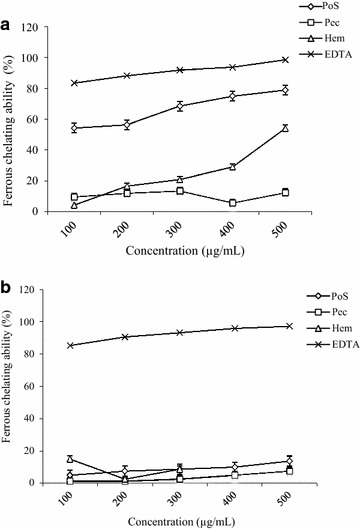
Ferrous ion chelating capacity (%) of polysaccharide isolate from *Allanblackia floribunda* stem bark (**a**) and *Chromolaena odorata* leaves (**b**)

Metal chelating capacity is important in reducing the concentration of transition metals that may act as catalysts to generate the first few radicals and initiate the radical-mediated oxidative chain reactions in biological systems. Ion-chelating agents may also inhibit the Fenton reaction and hydroperoxide decomposition. Fe^2+^ ion is the most powerful pro-oxidant able to form complexes with ferrozine. Earlier reports have revealed that Fe^2+^ accelerates lipid peroxidation by breaking down hydrogen and lipid peroxides formed by the Fenton free radical reaction. The chelating effect on ferrous ions is therefore a widely used method to evaluate antioxidant activity. The chelating ability of polysaccharide fractions are presented in Fig. 8. At the concentration of 100 to 500 µg/mL, PoS of *A. floribunda* presented the best activity (54.25 ± 4.07 to 78.91 ± 1 % (Fig. 8a). Pec of *A. floribunda* and the three fractions of *C. odorata,* showed poor ferrous ion chelating ability (Fig. 8a, b).

### Immunomodulatory activities of polysaccharide fractions

**Fig. 9:**
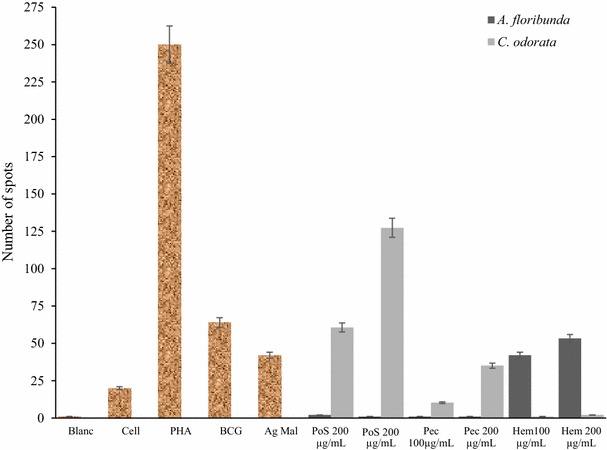
Effects of polysaccharide fractions on cell proliferation and interferon production by ELISPOT. Mal Ag: malaria antigen

The evaluation of cell proliferation and interferon production by ELISPOT in the presence of one mitogen (PHA) and two positive controls [BCG and malaria antigen (Mal Ag)] showed that PBMCs produced more interferon in the presence of mitogen and the two positive controls than in the negative control (Fig. 9). The ELISPOT test in the presence of the three polysaccharide fractions showed that not all extracts had an immunostimulatory capacity. In fact, PoS and Pec of *A. floribunda* showed poor stimulation in the production of INF-γ and proliferation of PBMCs (Fig. 9). In this specie, Hem produced significant quantity of spots (Fig. 9). At 200 µg/mL, *A. floribunda* Hem produced more spots (63 ± 5) than BCG (41 ± 2.3 spots) and Mal Ag (32 ± 2.8 spots) at 50 µg/mL. In the case of *C. odorata,* the quantity of spots produced in the presence of PoS and Pec increased significantly with the concentration applied. C. *odorata* PoS produced 60.66 ± 5 and 127.33 ± 9 at 100 and 200 µg/mL respectively (47 % increase) while for C. *odorata* Pec, the increase was 28 %, 10.33–35 spots (Fig. 9).

## Discussion

Complementary and alternative medicines have been used historically around the world to treat many diseases. While the mechanisms of action of many plant products remain to be elucidated, further study in this area is essential for the identification of novel therapeutics with immunomodulatory activity [3]. The characterization of the various fractions by Bradford [20], Folin–Ciocalteu [21], and Dubois et al. [22] methods shows a variability of the different metabolites in plants. The presence of proteins can be justified by the presence of glycoproteins including AGPs. According to Chen et al. [29], the bioactivity of polysaccharides depends primarily on their chemical environment and their combination with other compounds such as proteins, polyphenols and lipids.

Looking at the saccharide composition of the three polysaccharide fractions, it was observed that these extracts had different monosaccharide compositions. In the PoS fractions, Ara and Gal were the major monosaccharides found, supporting the fact that arabinogalactans are the primary macromolecules. Medicinal plants extracts containing AGPs have been shown to stimulate the proliferation and IgM-production in mouse lymphocytes [9, 30]. Pec fractions mostly contained GalA and Gal. The presence of GalA (up to 30 %) indicated the presence of soluble pectin, specifically the homogalacturonan polymers. Hem fractions contained predominantly Xyl, GalA and Xyl (upto 80 %) for *A. floribunda* and *C. odorata* respectively.

Natural antioxidants can be considered as potential therapeutic agents against many diseases resulting from oxidative stress such as diabetes, cancer, neurodegenerative and cardiovascular diseases. Four antioxidant assays, namely Ferric reducing antioxidant potential (FRAP), DPPH free radical scavenging activity, ABTS free-radical-scavenging and ferrous ion chelating ability were performed in order to explore the antioxidant potential. The DPPH and ABTS radical scavenging activities were tested to evaluate the ability of the polysaccharide fractions to provide hydrogen to a free radical. *A. floribunda* Hem and *C. odorata* PoS fractions showed the highest activity (over 60 % at 4 mg/mL). This activity could be due to the large amount of phenolic compounds found in these fractions. Polyphenols have been previously identified in the polysaccharide fractions of various plants [31, 32], and it has been reported that polysaccharides are able to bind to polyphenols by intermolecular interactions [33]. The presence of polyphenols in polysaccharide fractions is therefore to be considered [8].

The immunostimulatory activity was perfomed through the proliferation of PBMCs and the production of INF-γ. The result showed that significant activity was observed in the Hem fraction of *A. floribunda* and PoS fraction of *C. odorata.* These fractions also showed the best DPPH and ABTS radical scavenging activity. For *C. odorata,* the presence of type II arabinogalactan in the PoS fraction could justify these properties [3, 34]. Although, *A. floribunda* PoS are rich in type II arabinogalactan, this fraction did not show immunomodulatory property. However, the results obtained with Hem of *A. floribunda* are in agreement with those of Zha et al. [35]. These authors showed that, hemicellulose HPS-1B23 isolated from the stems of *Dendrobium huoshanense*, a plant used in traditional Chinese medicine, stimulates the immune system at different levels [stimulation of interferon gamma (IFN-γ) and the secretion of tumour necrosis alpha factors (TNF-α)].

## Conclusion

In this study, soluble polysaccharides, pectic and soluble hemicellulose fractions were isolated from the dry stem bark of *A. floribunda* and the leaves of *C. odorata* used in Cameroonian traditional medicine. An evaluation of their chemical and biological properties showed that *A. floribunda* Hem fraction and *C. odorata* PoS fraction had significant DPPH and ABTS radical scavenging activities and immunostimulatory activity via stimulation of PBMC and production of IFN-γ in a dose-dependent manner. These results may justify the traditional use of these plants. Further research is necessary to have supporting evidence that the antioxidative and immunomodulative activities of these fractions are really connected to the polysaccharides and not polyphenols.

## Abbreviations

AGPs: arabino galactan proteins
PBMCs: peripheral blood mononuclear cells
ELISPOT: enzyme linked immunospot technique
CWM: cell wall material
DPPH: 1,1-diphenyl-2-picryl hydrazyl
ABTS: acide 2,2′-azino-bis-3-éthylbenzylthiazoline-6-sulphonique
RPMI medium: Roswell Park Memorial Institute medium
Dw: dry weight
